# Fate of Zinc Oxide Nanoparticles Coated onto Macronutrient Fertilizers in an Alkaline Calcareous Soil

**DOI:** 10.1371/journal.pone.0126275

**Published:** 2015-05-12

**Authors:** Narges Milani, Ganga M. Hettiarachchi, Jason K. Kirby, Douglas G. Beak, Samuel P. Stacey, Mike J. McLaughlin

**Affiliations:** 1 Soil Science, School of Agriculture, Food & Wine, University of Adelaide, Glen Osmond, Australia; 2 Department of Agronomy, Throckmorton Plant Sciences Centre, Kansas State University, Manhattan, Kansas, United States of America; 3 CSIRO Land and Water Flagship, Environmental Contaminant Mitigation and Technologies Program, Advanced Materials Transformational Capability Platform (Nanosafety), Waite Campus, Glen Osmond, Australia; Institute for Materials Science, GERMANY

## Abstract

Zinc oxide (ZnO) nanoparticles may provide a more soluble and plant available source of Zn in Zn fertilizers due to their greater reactivity compared to equivalent micron- or millimetre-sized (bulk) particles. However, the effect of soil on solubility, spatial distribution and speciation of ZnO nanoparticles has not yet been investigated. In this study, we examined the diffusion and solid phase speciation of Zn in an alkaline calcareous soil following application of nanoparticulate and bulk ZnO coated fertilizer products (monoammonium phosphate (MAP) and urea) using laboratory-based x-ray techniques and synchrotron-based μ-x-ray fluorescence (μ–XRF) mapping and absorption fine structure spectroscopy (μ–XAFS). Mapping of the soil-fertilizer reaction zones revealed that most of the applied Zn for all treatments remained on the coated fertilizer granule or close to the point of application after five weeks of incubation in soil. Zinc precipitated mainly as scholzite (CaZn_2_(PO_4_)_2_.2H_2_O) and zinc ammonium phosphate (Zn(NH_4_)PO_4_) species at the surface of MAP granules. These reactions reduced dissolution and diffusion of Zn from the MAP granules. Although Zn remained as zincite (ZnO) at the surface of urea granules, limited diffusion of Zn from ZnO-coated urea granules was also observed for both bulk and nanoparticulate ZnO treatments. This might be due to either the high pH of urea granules, which reduced solubility of Zn, or aggregation (due to high ionic strength) of released ZnO nanoparticles around the granule/point of application. The relative proportion of Zn(OH)_2_ and ZnCO_3_ species increased for all Zn treatments with increasing distance from coated MAP and urea granules in the calcareous soil. When coated on macronutrient fertilizers, Zn from ZnO nanoparticles (without surface modifiers) was not more mobile or diffusible compared to bulk forms of ZnO. The results also suggest that risk associated with the presence of ZnO NPs in calcareous soils would be the same as bulk sources of ZnO.

## Introduction

Zinc (Zn) deficiency is one of the most common micronutrient problems that adversely affects agricultural production, particularly in alkaline calcareous soils [1]. Calcareous soils constitute a major resource for agricultural use, mainly localized in arid or Mediterranean environments of the world [2]. The most important soil parameters that limit Zn availability to plants in calcareous soils are the alkaline pH, which reduces Zn solubility, and the high calcium carbonate (CaCO_3_) content, which can adsorb and precipitate Zn [3, 4]. Inorganic sources of Zn such as zinc oxides (ZnO) and zinc sulphates (ZnSO_4_ H_2_O or ZnSO_4_ 7H_2_O) are commonly being used as Zn fertilizers to correct Zn deficiency in soils [5]. The effectiveness of applied Zn fertilizers is strongly correlated with the solubility of the Zn source [6, 7], which is heavily influenced by the properties of the soil to which it is applied.

Solubility and dissolution kinetics of particles depend on their surface area. Therefore, the rate and extent of dissolution is greater for nanoparticles compared to bulk materials [8] due to their smaller particle sizes, higher specific surface area and an increased proportion of reactive surface atoms [9, 10]. It follows then, that the use of zinc oxide nanoparticles (ZnO NPs) in Zn fertilizers may increase Zn dissolution and consequently its bioavailability in problematic soils, such as calcareous soils. Diffusion of dissolved Zn is the main mechanism for the movement of Zn from fertilizer to the plant roots following the dissolution process [11]. A small increase in the diffusion radius of Zn in soil following the application of ZnO NPs may also considerably increase the volume of the Zn-enriched soil and the subsequent availability of Zn fertilizer to plants. Therefore, use of nanoparticulate sources of Zn in Zn fertilizers may increase Zn use efficiency and reduce the quantity and frequency of Zn fertilizer application.

Despite the benefits speculated for the application of ZnO NPs as a source of Zn in soil, nanoparticles are unlikely to remain in their original form following incubation in soils [12]. Soil components will inevitably interact with released ZnO nanoparticles in the soil and affect the spatial distribution and speciation of added Zn. Although application of ZnO NPs as a source of Zn aims to optimize efficiency of applied Zn fertilizer, it is the fate and behaviour of ZnO NPs in soils that will ultimately determine its effectiveness and/or environmental risk (e.g. increased mobility and toxicity of ZnO NPs). The chemical and physical behaviour of soluble and bulk sources of Zn in soils have been widely investigated [13–18]. There is limited understanding of the fate and transformations of ZnO NPs in natural soils, especially calcareous soils. Scheckel *et al*. (2010) [19] examined the reactions of nanoparticulate ZnO with suspensions of kaolin and found that nanoparticles transformed very rapidly (~1 day) to soluble and sorbed Zn. However, no comparison was made with bulk-sized ZnO particles. A previous study showed that the kinetic of Zn dissolution from ZnO coated fertilizers in a porous media was not affected by the size of ZnO particles used for coating [20]. However, in natural soil environments soil properties such as presence of organic materials in soil, the ionic strength of soil solution, soil pH and minerals type and content can affect mobility and bioavailability of ZnO nanoparticles [21]. The information on the reactions and solid phase speciation of Zn following the addition of ZnO NPs to soils is of vital importance for a realistic assessment of potential effectiveness and/or adverse effects of using ZnO nanoparticle-coated fertilizers in Zn deficient soils.

Although the complexity of soil systems and heterogeneity at the micro-scale were previously obstacles in solid phase speciation of soils, synchrotron based x-ray absorption spectroscopy (XAS) techniques have provided an excellent research tool for *in situ* investigations at the molecular level of the solid phase speciation of elements such as Zn in soil environments [22–24]. The sensitivity of μ-focused XAS techniques in the complex local micro-structural environment of the element of interest and low detection limits give them an advantage over other physical techniques [23]. Synchrotron x-ray florescence (XRF) analysis also allows quantitative distribution mapping of several elements simultaneously and with high resolution, in complex environmental systems [25].

The aim of this study was to examine the diffusion and solid phase speciation of Zn in an alkaline calcareous soil following application of nanoparticulate and bulk ZnO coated fertilizer products (monoammonium phosphate (MAP) and urea). A fertilizer diffusion cell method and synchrotron-based μ-x-ray fluorescence (μ–XRF) mapping and absorption fine structure spectroscopy (μ–XAFS) were used to examine the diffusion and speciation of Zn treatments in the selected calcareous soil. The information will be used to assess the efficacy and environmental risk of ZnO NP coated fertilisers in calcareous soils.

## Materials and Methods

### Characterisation of ZnO particles and preparation of coated fertilizers

**Fig 1:**
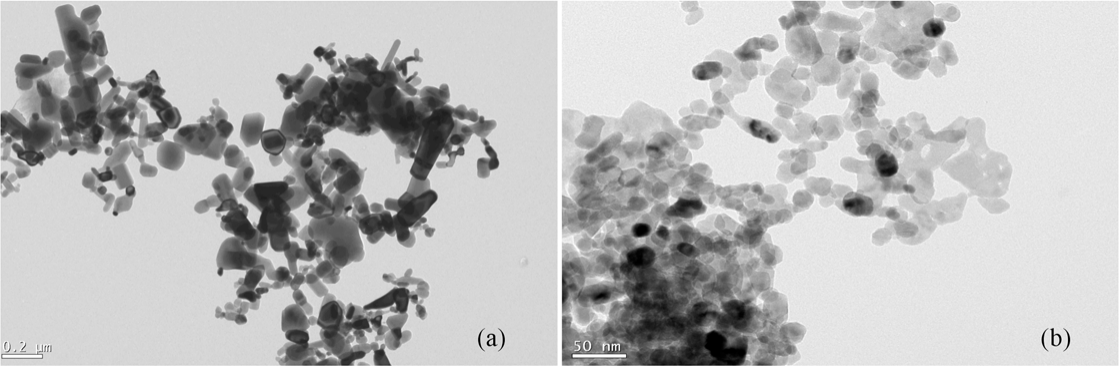
Transmission electron microscopy (TEM) images of (a) bulk ZnO (nominal diameter less than 1 micro-meter) and (b) ZnO nanoparticles (nominal size of 20 nm).

Commercial ZnO nanoparticle powder was purchased from Nanostructure & Amorphous Material Inc. (Houston, USA) with a nominal particle size of 20 nm. Bulk ZnO powder (99.9%, nominal diameter < 1 μm) was purchased from Sigma-Aldrich (Sydney, Australia). Surface capping agents or modifiers were not present on ZnO particles. The methods used to characterize primary nanoparticulate and bulk ZnO particles and their findings can be found in Milani *et al*. (2012) [20]. The ZnO powders were examined using transmission electron microscopy (TEM; Phillips CM200, Edindhoven, The Netherlands) (Fig 1) and x–ray diffraction (XRD) (PANalytical X’Pert Pro, Almelo, The Netherlands) techniques. The XRD patterns were used to confirm the presence of ZnO in samples and to estimate crystallite size of particles. The Brunauer-Emmett-Teller (BET) surface area equation [26] after liquid N_2_ adsorption (Quanta Chrome, USA) was used to determine specific surface area of particles. The crystallite structure determined using XRD patterns showed that the ZnO NPs were exclusively composed of zincite (zinc oxide), while the XRD pattern of bulk ZnO revealed a dominant presence of zincite crystals along with minor amounts of hydrozincite. The crystallite size estimates of ZnO NPs based on the Sherrer equation using full width at half maximum of the XRD pattern and BET-N_2_ analysis suggested that the size of ZnO NPs are consistent with the nominal size provided by the manufacturer (20 nm). BET-N_2_ analysis on ZnO powders resulted in surface areas equal to 31 m^2^g^-1^ and 12 m^2^g^-1^ for ZnO NPs and bulk ZnO, respectively.

The monoammonium phosphate (MAP- Mosaic Co., Plymouth, USA) and commercial urea fertilizer granules were coated with ZnO powders using the method described in Milani *et al*. (2012) [20]. The four coated fertilizer treatments were MAP granules coated with nanoparticulate ZnO (NanoMAP), MAP granules coated with bulk ZnO (BulkMAP), urea granules coated with ZnO NPs (NanoUrea) and urea granules coated with bulk ZnO (BulkUrea).

**Fig 2:**
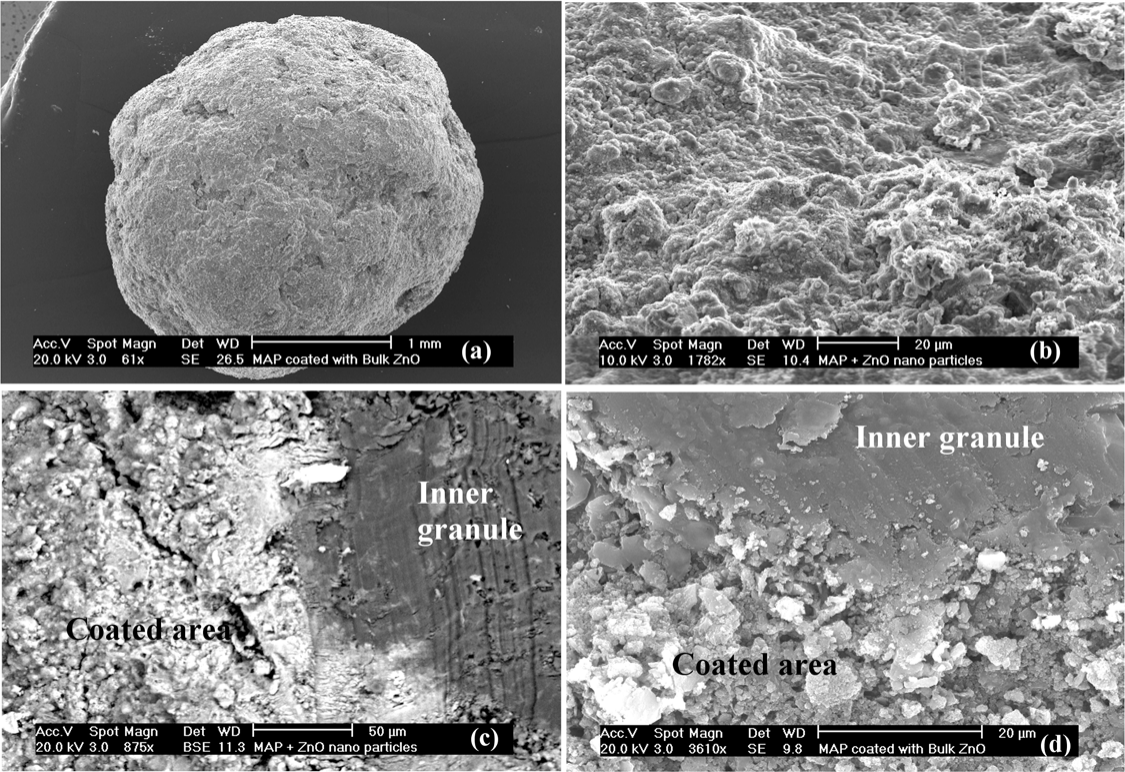
Scanning electron microscopy (SEM) images of (a) NanoMAP granule, (b) distribution of ZnO nanoparticles at the surface of NanoMAP granule, (c) cross-sectioned NanoMAP granule illustrating the core of MAP granule in dark grey and coated surface with ZnO nanoparticles in light grey in backscatter mode and (d) cross-sectioned BulkMAP granule showing inner granule and rough coated surface with bulk ZnO particles.

**Fig 3:**
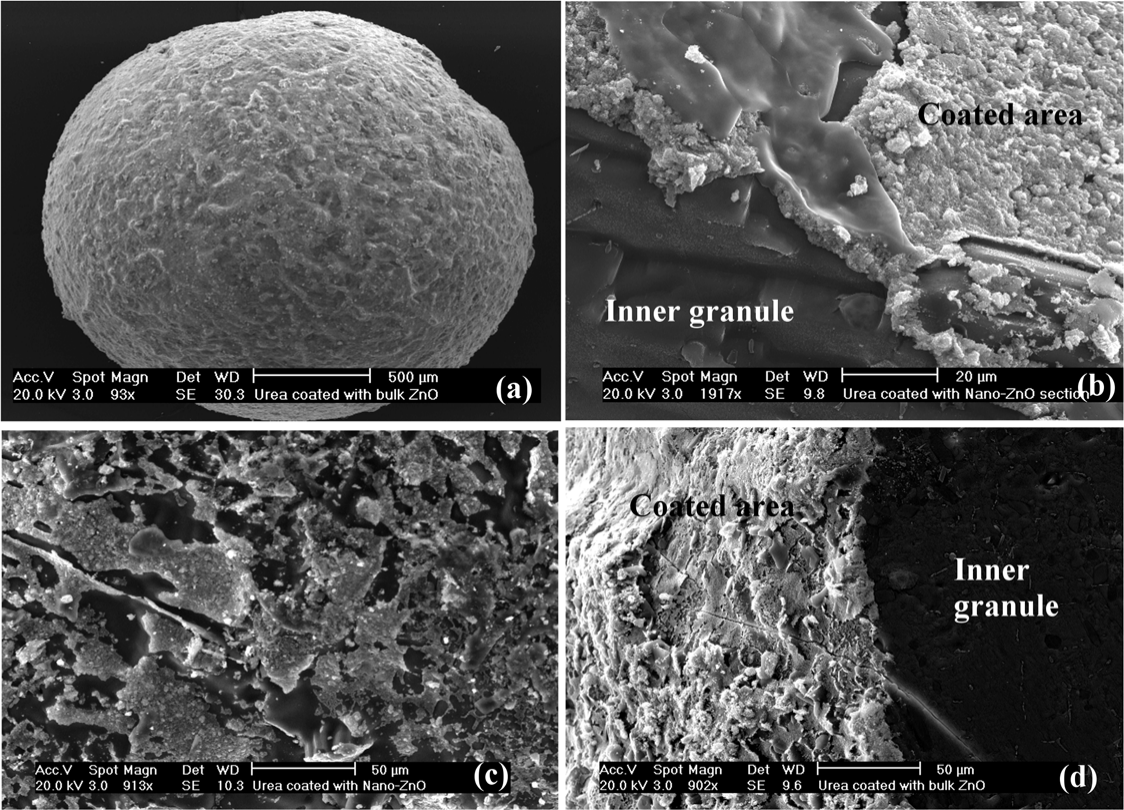
Scanning electron microscopy (SEM) images of (a) urea granule coated with bulk ZnO, (b) cross-sectioned NanoUrea granule representing inner urea granule and coated surface of the granule with ZnO nanoparticles, (c) surface of NanoUrea granule showing distribution of ZnO nanoparticles at the surface of urea granule and (d) cross-sectioned BulkUrea granule illustrating coated surface of urea granules with bulk ZnO and also inner urea granule in dark grey.

The distribution of ZnO powders on the surface of the coated fertilizer granules was investigated using scanning electron microscopy (SEM; Philips XL30 field emission SEM, Eindhoven, The Netherlands). Fertilizer granules with/without ZnO coating were cross sectioned, coated with carbon and gold to reduce static electric charge accumulation and finally mounted onto aluminium specimen holders for SEM analysis. Elemental compositions of selected points of interest at the surface of fertilizer granules as well as the points in the core of the granules (after fracture) were examined using integrated energy dispersive X-ray analysis (EDXA; Genesis EDX Spectrometer system, NJ, USA). Mineralogy of the coated fertilizer granules was investigated using XRD analysis. The methods used and their findings can be found in Milani *et al*. (2012) [20]. In summary, SEM images illustrated a nearly homogeneous distribution of the coating at the surface of the fertilizer granules (Fig 2 and Fig 3). The EDXA spectra of MAP granules coated with ZnO powders showed that the coatings of granules mainly consisted of phosphorus (P), Zn and oxygen (O) followed by nitrogen (N), whereas the elemental composition of inner granule were predominantly P, O and N (S1, S2 and S3 Figs). The elemental composition at the surface of commercial urea fertilizers and the core of urea granules coated with ZnO mainly consisted of N and O (S4 and S5 Figs). The surface of urea granules coated with ZnO predominantly contained Zn followed by O (S5 and S6 Figs). Mineralogical characterization using XRD patterns showed dominant and intense peaks of zinc ammonium phosphate (Zn(NH_4_)PO_4_) for coated MAP fertilizers and a small amount of zincite. In contrast, XRD patterns of coated urea granules revealed the Zn as the mineral zincite at the surface of the fertilizer granule. The XRD patterns showed that ZnO NPs at the surface of urea granules had a similar crystallite size (20–30 nm) to the added primary ZnO NPs.

### Synchrotron based μ-XRF mapping and μ-XAFS data collection and analysis

Zinc distribution from ZnO-coated fertilizers in an alkaline calcareous soil was investigated following their incubation in soil using μ-XRF mapping. Solid phase speciation of selected points on, or adjacent to, the incubated fertilizer granules in the soil was also studied using μ-XAFS. The fertilizer diffusion protocol developed by Hettiarachchi *et al*. (2008) [22] was adopted for use in this study. A highly calcareous sandy loam soil [Sodic Calcixerept [27]] was collected from upper Eyre Peninsula, South Australia to be used in the experiment. Selected physical and chemical properties of the soil are presented in Table 1.

**Table 1:** Selected physical and chemical properties of the soil.

Soil property	value
pH (1:5 soil:water)	8.4
Electrical Conductivity (dS m^-1^)	0.13
Cation Exchange Capacity (cmol(+) kg^-1^)	7.0
Carbonates (g kg^-1^)	357
Clay (g kg^-1^)	140
Silt (g kg^-1^)	37.0
Sand (g kg^-1^)	824
Organic C (g kg^-1^)	6.0
Total Zn (mg kg^-1^)	16.0
Total P (mg kg^-1^)	330
Total Fe (mg kg^-1^)	7390

Four experimental cells (5 cm long x 5 cm wide x 0.5 cm deep Plexiglass holders with 25 mm diameter circular Kapton windows at the top) were uniformly packed with 17 g of slightly wetted soil and further wetted to 60% of its maximum water holding capacity [28] by adding ultra-pure deionised water. The cells were equilibrated for 24 hr and then one coated MAP granule or 5 coated urea granules were placed into the centre of each experimental cell close to the Kapton window (centre top). Experimental cells were then incubated in a temperature controlled environment at 25°C for five weeks under aerobic condition. The individual cells were weighed and sufficient ultrapure deionised water added during the experiment to maintain the soils at 60% of maximum water holding capacity.

The μ-XRF mapping and μ- XAFS data collection were performed at beamline 13-BM-D (GeoSoilEnviro Consortium of Advanced Radiation Sources), at the Advanced Photon Source at Argonne National Laboratory, Argonne, IL. The electron storage ring is operated at 7 GeV and 13-BM is a bending magnet beamline equipped with a Si (111) monochromator with an energy range of 6–28 keV. Experimental cells containing fertilizer-incubated soils were mounted onto the sample stage on the rotation axis of an *x-y-θ* stepping motor stage with the side containing Kapton x-ray window facing the beam. The incident x-ray beam was focused to a 50 μm spot size to assess elemental distribution in the soil as a function of distance from point of fertilizer granule application. The μ-XRF mapping data was collected at ambient temperature using a solid-state energy-dispersive x-ray detector that allowed simultaneous detection of fluorescence signals from multiple elements. At each position, the fluorescence signal from a given element was proportional to the integrated number of atoms of that element along each transect of synchrotron beam. Florescence data were collected for a 7000 by 4800 μm area for the soil sample treated with NanoMAP granules, a 7000 by 5000 μm area for the soil sample treated NanoUrea and a 5000 by 2950 μm area for soil sample treated with BulkUrea fertilizer granules (The μ-XRF map for the soil treated with BulkMAP fertilizer granule was previously collected by Hettiarachchi *et al*., 2008). Micro-XRF maps for Ca, Cu, Fe, Mn, Ti and Zn were collected. Micro-XRF maps of Ca and Ti were used to identify the location of fertilizer granules in petri dishes. For each μ-XRF map, 2 or 3 points of interest (determined based on highest relative intensity) were identified for μ–XAFS speciation analysis.

The μ- XAFS spectra of selected points were collected from 150 eV below to 640 eV above the K-edge of Zn (9659 eV) in fluorescence mode using a 13-element Ge solid-state detector. Three replicate scans from selected points on each fertilizer treatments as well as unexposed fertilizer granules were collected. In addition, μ- XAFS of ZnO powders and standard Zn compounds were collected in the same mode (fluorescence) and scan conditions, after diluting standard compounds using boron nitride (BN) to bring concentration of Zn in each Zn standard to approximately 0.5%. Zn standard compounds included ZnSO_4_.7H_2_O, Zn(OH)_2_, Zn(NH_4_)PO_4_.6H_2_O, smithonite (ZnCO_3_), zincite (ZnO), hydrozincite (Zn_5_(CO_3_)_2_(OH)_6_), scholzite (CaZn_2_(PO_4_)_2_.2H_2_O), franklinite (Zn_0.6_Mn_0.3_(II)Fe_0.1_(II)Fe_1.5_(III)Mn_0.5_(III)O_4_), hopeite (Zn_3_(PO_4_)_2_.4H_2_O), willemite [Zn_2_(SiO_4_)], ferrihydrite (Fe_5_HO_8_.4H_2_O)-adsorbed Zn and calcite-adsorbed Zn.

The collected μ-XAFS spectra were analysed using the Athena software package [29]. Replicate scans of each sample were aligned using a reference spectrum (Zn foil, 7659 eV) and then averaged into a final spectrum for each sample. Averaged spectra were background-corrected and normalized. Linear combination fitting (LCF) operations were also performed using Athena. Since the accuracy of the LCF species identified is limited by the choice of standard compounds and the presence of impure minerals in soil [23], a combination of techniques (x-ray spectroscopic, microscopic and diffraction) were used to identify the reaction products of unexposed fertilizers and validate the reaction product identification.

## Results and Discussions

### Zinc diffusion and spatial distribution

**Fig 4:**
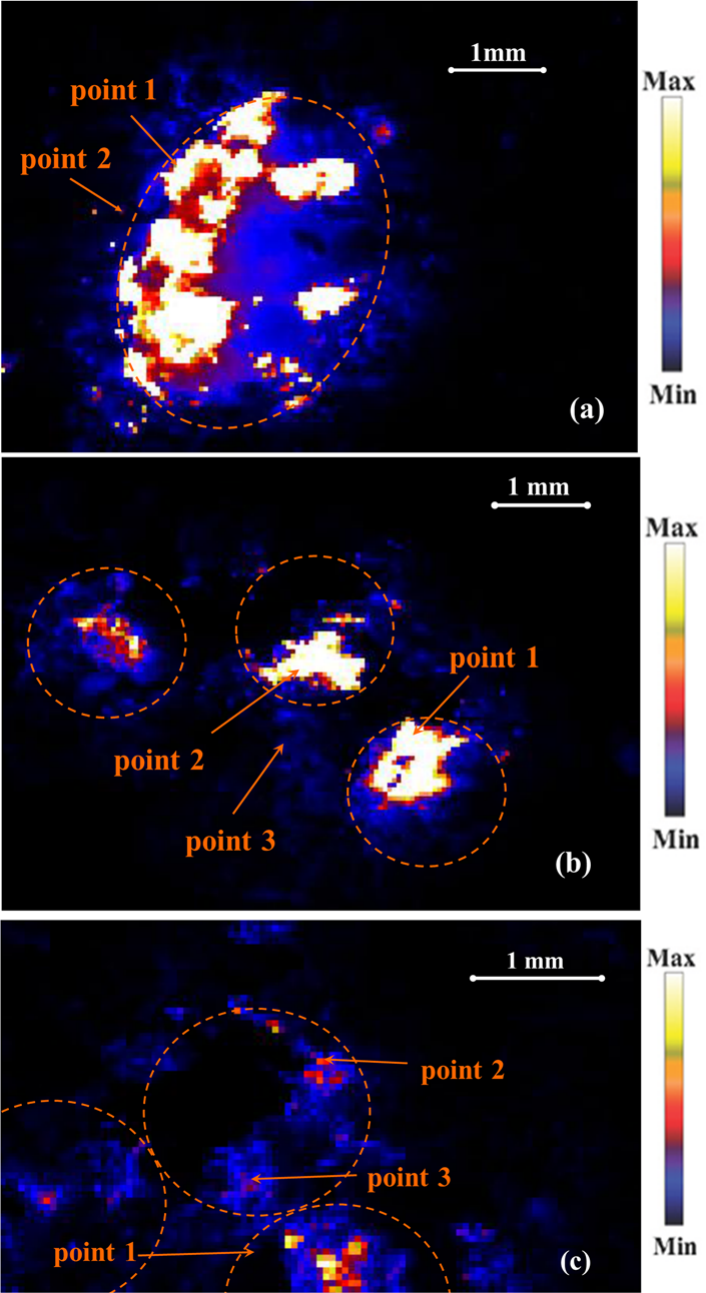
Micro-XRF maps of Zn for soil incubated with (a) NanoMAP granule, (b) NanoUrea granule and (c) BulkUrea granules. The colour scheme represents white-yellow for high concentrations and blue-black for low concentrations of the elements. The dashed area represent the probable location of the fertilizer granules in the soil sample and the marked points in Zn Kα map indicate the locations for which μ-XAS spectra were collected.

Micro-XRF maps showing spatial distribution of Zn on and around coated fertilizer granules incubated in the soil can be found in Fig 4. The μ–XRF map of the NanoMAP granule revealed that most of the fertilizer Zn remained at the surface of the granule after five weeks of incubation. The observed high intensity of elements on one side of the MAP granule (Fig 4A) may be due to the granule being partially covered with soil and the fluorescent signal being lost in the concealed regions. Moreover, an artifact caused by the offset of the x-ray beam angle on the curved granule might have resulted in a higher intensity of elements in one side of the granule. The μ–XRF maps of Zn treatments for the coated urea granules can be found in Fig 4B and Fig 4C. Although complete dissolution of urea granules made it difficult to recognize their accurate position or size in the scanned region, dashed areas illustrate the likely original location of the coated urea granules in the scanned area of experimental cells found *via* overlapping maps of different elements. As observed for MAP granules, Zn released from the coating of urea granules mainly remained close to the application point of granules.

Limited diffusion of Zn from coated fertilizers may be due to reactions of ZnO NPs at the surface of granule, mass flow of water in soil towards the fertilizer granules or pH effects. Transformation of nanoparticulate ZnO in the coating of MAP granules to zinc phosphate-like species may have hindered expected diffusion of ZnO NPs in the calcareous soil. This process resulted in the same restricted Zn diffusion which was previously reported for commercial Zn-enriched MAP granules (with Zn incorporated throughout the granule) [22, 30]. Although ZnO in the coating of urea granules did not transform to other Zn species, aggregation of released ZnO NPs around the granule due to high pH and ionic strength would be expected in soil solution adjacent to the dissolved urea granule[21]. This process may have reduced dissolution and subsequent diffusion of Zn in the soil.

Mass flow of water from the soil towards the hygroscopic fertilizer granule in the opposite direction to Zn diffusion could be a probable mechanism restricting Zn diffusion the same way as it was earlier reported for restricted diffusion of phosphorus in a highly calcareous soil [31]. Acidity produced by the fertilizer granule may also affect the solubility, speciation and diffusion of Zn in the soil. Suspensions of coated urea fertilizer granules in ultra-pure deionised water (Millipore) had a pH of *ca*.7.5 whereas pH of coated MAP suspension was *ca*. 4.8 [20]. Therefore, solubility and reaction products of Zn from the coating of MAP and urea granules in the soil would be affected by the acidity produced by the fertilizer granule. The initial neutral to alkaline pH in the soil around coated urea granules may have reduced solubility of ZnO NPs. In comparison, MAP fertilizer granules reduced the pH of the environment adjacent to the granule [32]. Despite this decrease in soil pH which may promote dissolution of ZnO to Zn^2+^, the large pH buffering capacity of the alkaline calcareous soil may limit the solubility and diffusion of Zn around the MAP granules[33]. Moreover, phosphate dissolved from the MAP granule could rapidly reduce the release of Zn^2+^ as a result of precipitation of Zn phosphate-like species adjacent to the MAP granule [12].

### Solid phase speciation of Zn

**Fig 5:**
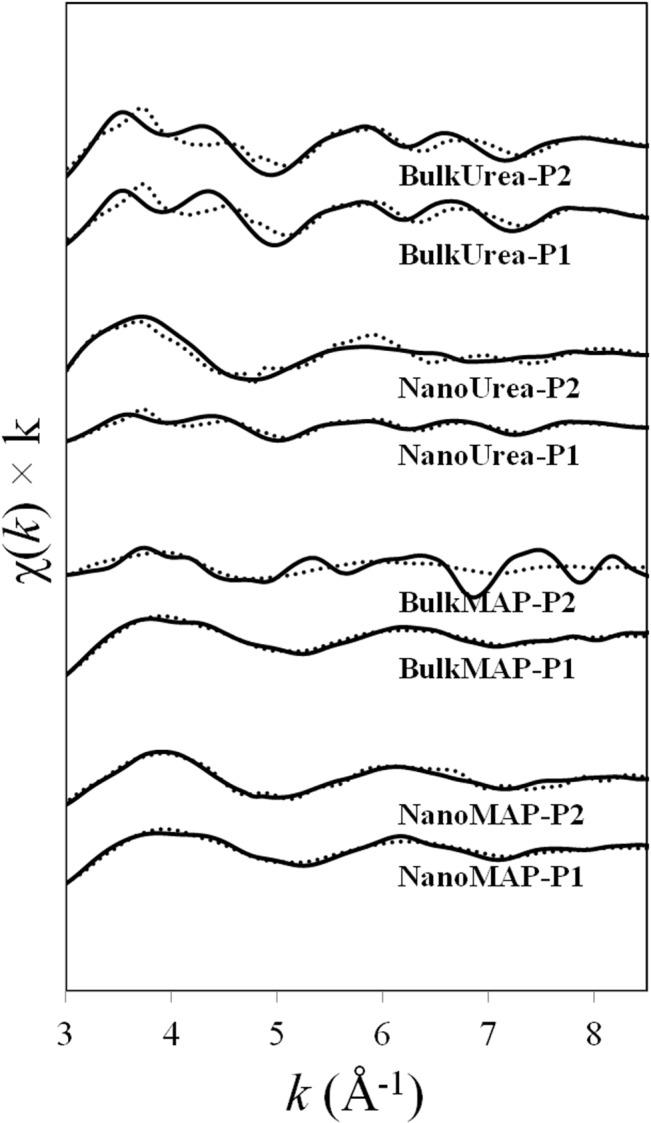
The k-weighted (χ(k)) x-ray absorption fine structure (XAFS) spectra of selected points of interest in Fig 2 (solid lines) and related linear combination fits (dotted lines).

The Zn K-edge XAFS spectra collected from unexposed fertilizer granules as well as selected points of interest on, or adjacent to, the MAP and urea fertilizer granules incubated in the calcareous soil were analysed using Linear Combination Fitting (LCF) method. The Zn K-edge XAFS spectra and fitted lines can be found in Fig 5. The resultant Zn speciation for each point based on the LCF method is listed in Table 2.

**Table 2:** Relative proportion of Zn species at points of interest on coated fertilizer granules incubated in soil and unexposed coated fertilizer granules determined by linear combination fittings of x-ray absorption fine structure (XAFS) spectra.

Treatments	Zn(NH_4_)PO_4_	Scholzite	Zincite	Hydrozincite	Smithonite	Zn(OH)_2_	χ^2^_red_ ^a^
NanoMAP (P1)	43	47	-	-	-	9	0.038
NanoMAP (P2)	54	-	-	-	46	-	0.537
BulkMAP (P1)	48	34	-	12	-	6	0.028
BulkMAP (P2)	-	75	-	-	25	-	0.447
NanoMAP(unexposed)	22	63	0	-	-	15	0.143
BulkMAP(unexposed)	19	63	6	-	-	12	0.177
NanoUrea (P1)	-	-	62	-	11	27	0.054
NanoUrea (P2)	-	-	66	-	-	34	0.051
NanoUrea (P3)	-	-	11	-	-	89	0.205
BulkUrea (P1)	-	-	57	-	14	29	0.363
BulkUrea (P2)	-	-	50	-	25	25	0.506
BulkUrea (P3)	-	-	42	-	9	50	0.325
NanoUrea(unexposed)	-	-	100	-	-	-	1.370
BulkUrea(unexposed)	-	-	93	7	-	-	0.070

^a^ χ^2^_red_ (reduced chi square) = [Σ(fit—data)^2^ / σ^2^]/(N_data_—N_components_-1), where σ^2^ is the known variance of fits, N_data_is the number of data points and N_components_ is the number of components in the fit. As indicated, reduced chi square (χ^2^_red_) reported by the Athena software is a measure of the sum of squares of the final misfits (see Athena Users’ Manual for details).

Similar Zn speciation was found for unexposed NanoMAP and BulkMAP granules which was predominantly scholzite (more than 60%), followed by Zn(NH_4_)PO_4_ and Zn(OH)_2_. Minor amount of zincite was found in unexposed ZnO-coated MAP granules suggested transformations of ZnO as a result of the reactions with phosphate in MAP fertilizer granules during the manufacturing process. The x-ray diffraction patterns identified Zn(NH_4_)PO_4_ as the major Zn species at the surface of unexposed MAP fertilizers. According to the μ-XAFS analysis, the major Zn species present were scholzite (CaZn_2_(PO_4_)_2_ 2H_2_O) and Zn(OH)_2_-like species. Considering that the XRD technique provides information on the structure of crystalline substances [34], these results suggest that scholzite and Zn(OH)_2_ identified using μ-XAFS technique were in amorphous or poorly crystalline forms which were not detected by XRD. The Zn species found in unexposed urea granules coated with ZnO NPs was exclusively zincite (ZnO). Unexposed urea granules coated with bulk ZnO contained minor amounts (7%) of hydrozincite (Zn_5_(CO_3_)_2_(OH)_6_) in addition to zincite species (Table 2) which can be attributed to the minor amounts of hydrozincite in bulk ZnO powder. Zinc oxide particles coated at the surface of urea granules may also transform to hydrozincite species at the atmospheric partial pressure of CO_2_ (10^–3.5^) during coating and handling processes [35].

A comparison of μ-XAFS spectra of the points of interest at the surface of coated MAP granules incubated in soil and unexposed MAP fertilizer granules indicated a reduction in the proportion of scholzite species and increase in the percentage of Zn(NH_4_)PO_4_ species with incubation in soil (Table 2). For example, μ-XAFS at point P1 at the surface of a NanoMAP granule incubated in soil (Fig 4A), showed Zn speciation to be 43% Zn(NH_4_)PO_4_ and 47% scholzite. On the other hand, the relative proportion of Zn(NH_4_)PO_4_ and scholzite were 23% and 62% at the surface of an unexposed NanoMAP granule, respectively (Table 2). The same trend was observed for BulkMAP granules where the relative proportion of Zn(NH_4_)PO_4_ increased from 19% at the surface of unexposed BulkMAP granules to 48% at the surface of granules incubated in the soil; and the percentage of scholzite decreased from 63% in unexposed BulkMAP granules to 34% at point P1 at the surface of BulkMAP granules incubated in the soil (Table 2). The solubility constant for scholzite (log *K*_sp_ = -9.46[36]) and Zn(NH_4_)PO_4_ (log *K*_sp_ = -12.4; Degryse *et al*., unpublished data) at 0.00038 atm pressure for CO_2_(g) (which is the probable CO_2_ pressure for calcareous soils) indicate that Zn(NH_4_)PO_4_ is more soluble (less stable) than scholzite in the experimental conditions investigated. An enhanced percentage of more soluble Zn species suggests dissolution of Zn at the surface of incubated MAP granules and re-precipitation in the ion saturated region immediately at the surface of the fertilizer. With increasing distance from the coated MAP granules, significant amounts of Zn carbonate species were identified (Table 2). Solid phase speciation at points P2, adjacent to the NanoMAP (Fig 4A) and BulkMAP granules incubated in the soil indicated Zn species to be 46% and 25% smithonite (ZnCO_3_), respectively (Table 2). Earlier investigations of the reaction products of ZnSO_4_ plus ammonium phosphate (NH_4_H_2_PO_4_) in calcareous soils has also confirmed formation of Zn(NH_4_)PO_4_, Zn_3_(PO_4_)_2_.4H_2_O and ZnCO_3_ species using XRD analysis [37]. In a synchrotron-based study, the dominant Zn species around a Zn-incorporated MAP granule in a calcareous soil were identified to be scholzite followed by minor amounts of willemite and zincite [22].

Zinc speciation at the surface of unexposed NanoUrea and BulkUrea granules consisted of 100% and 93% zincite, respectively (Table 2). However, solid phase speciation of points P1 and P2, at the point of application of the NanoUrea granules (Fig 4B), revealed formation of Zn(OH)_2_ species due to dissolution of ZnO NPs. The relative percentage of Zn species present as Zn(OH)_2_ increased to a dominant 89% at P3 (Fig 4B) which was the furthest point collected away from the NanoUrea granules in soil. In contrast, a combination of zincite, Zn(OH)_2_ and smithonite species were found for selected points in the soil incubated with BulkUrea fertilizer granules (Table 2). Gimbert *et al*. (2007) reported limited dissolution or partitioning of ZnO NPs in a suspension of an alkaline soil using flow field-flow fractionation. In contrast, our results suggested significant changes in Zn solid phase speciation of ZnO particles at the points on, or adjacent to, the coated urea granules due to dissolution of ZnO particles and re-precipitation of Zn(OH)_2_ and ZnCO_3_ species (Table 2). The stability of ZnO NPs in the Gimbert *et al*. experiment may be due the presence of a dispersant which could have educed/eliminated the dissolution and re-precipitation reactions of ZnO NPs in the soil suspension over the two weeks period of the experiment [38].

Investigations of the solid phase speciation of Zn added to calcareous soils have shown the importance of adsorption reactions on Fe- and Al-oxides at low added Zn concentrations [15, 39]. At elevated added concentrations of Zn in alkaline soils, adsorption sites can be saturated and chemical precipitation of Zn as Zn(OH)_2_, ZnCO_3_ and hydrozincite (Zn_5_(CO_3_)_2_(OH)_6_) are favoured over adsorption [13, 15]. In this study, significant amounts of Zn(OH)_2_ and ZnCO_3_ species were found at points on, or adjacent to, the coated urea fertilizer granules (Table 2). This suggests the formation of a Zn-rich region adjacent to the fertilizer granule due to dissolution of ZnO coated at the surface of the granule. High Zn concentrations in the vicinity of coated fertilizer granules likely favour precipitation reactions over adsorption reactions and therefore adsorbed Zn species were not detected by solid phase speciation of Zn in this soil. Hettiarachchi et al. (2008) also reported the absence, or very insignificant amounts, of adsorbed Zn species around the MAP-Zn granules in a calcareous soil [22].

**Fig 6:**
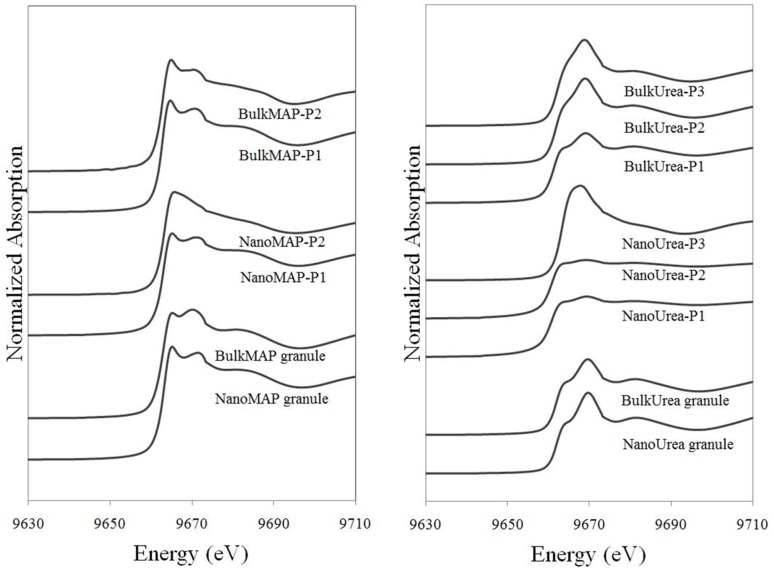
The normalized Zn micro-x-ray absorption near-edge structure (μ-XANES) spectra collected at points of interest on and around the coated MAP fertilizer granules (left) and coated urea fertilizer granules (right) incubated in a highly calcareous soil. Micro-XANES spectra collected from unexposed coated fertilizer granules also illustrated in the relevant graphs.

The μ-XANES spectra region of selected points for the soil treated with MAP and urea granules are shown in Fig 6. Careful visual observations of the μ-XANES spectra in Fig 6 together with the μ-XRF maps in Fig 4 suggested that μ-XAFS spectra of NanoUrea P1 and P2 might have been affected by self-absorption effects. Although the highest concentration of Zn was assumed to be *ca*.1000 mg kg^-1^ for MAP-Zn granules [22], it is possible that these two spots were more concentrated than 1000 mg kg^-1^. Therefore, it should be noted that the conclusion that these spots were zincite and/or zinc hydroxide is tentative. Self- absorption effects were not observed for all other points including NanoUrea P3. Visual observation of MAP spectra demonstrated similar Zn speciation for MAP granules treated with bulk ZnO or ZnO NPs. Spectra of urea granules illustrated more obvious changes in the shape and structure in comparison to the spectra collected from MAP treatments, indicating greater reactivity of Zn species at the surface of urea granules.

## Conclusions

This study provides better understanding of the diffusion and transformation process of ZnO nanoparticles coated onto macronutrient fertilizers *in situ* in soil. It also highlights changes in solid phase speciation of Zn from ZnO nanoparticles associated with macronutrient fertilizers in a natural soil environment. Micro–XRF mapping showed that diffusion of Zn from the coated granules was highly restricted, irrespective of whether the bulk or nanoparticulate forms of ZnO was used. The μ-XAFS data suggested that ZnO-coated MAP and urea fertilizer granules produce different reaction products following incubation in the calcareous soil used. Chemical reactions of Zn sources with phosphate species in the MAP granule affected the solid phase speciation of Zn in the soil. Transformation of ZnO to Zn(NH_4_)PO_4_ and scholzite species in coated MAP granules hindered expected high solubility of nanoparticulate ZnO. Although ZnO particles remained as zincite at the surface of coated urea granules, high pH and ionic strength in soil solutions from hydrolysis of urea would have promoted aggregation of any ZnO NPs released from the granules and masked the effect of particle size on solubility and mobility of ZnO particles. Therefore, significant greater dissolution of ZnO NPs was not observed for NanoUrea granules compared to BulkUrea granules as was anticipated based on theoretical considerations [8].These results are in agreement with our previous study on dissolution kinetics of the same fertilizer granules in porous media, which showed size of ZnO particles did not affect solubility and dissolution kinetics of Zn from coated fertilizer granules [20]. Similar dissolution rate and equilibrium solubility for bulk and nanoparticulate ZnO due to aggregation of ZnO NPs was previously reported [40]. Our study suggests that the application of ZnO NPs (without surface modifiers) rather than bulk forms of ZnO as a micronutrient coating for urea or MAP appears to offer little benefit in terms of Zn dissolution and diffusion in calcareous soils. However, from an ecotoxicological point of view the risks associated with the presence of ZnO NPs in soils would be the same as bulk sources of ZnO.

## Supporting Information

S1 Dataset Micro-x-ray absorption fine structure (μ-XAFS) data for zinc standards and treatments used in the experiment. (XLSX)

S1 Fig The SEM-EDXA analysis of the surface of a commercial mono ammonium phosphate (MAP) fertiliser granule. This figure shows (a) scanning electron microscopy (SEM) image of the surface of commercial mono ammonium phosphate (MAP) fertiliser granule used in the experiment and (b) EDXA spectrum that is collected from the point at the surface of the granule indicated by a cross on the SEM image. Elemental composition of the point of interest is reported in the table.
(TIF)

S2 Fig The SEM-EDXA analysis of a cross sectioned MAP granule coated with ZnO nanoparticles in backscattered mode. The figure illustrates (a) coated area (left) and inner granule (right) as well as the spots from which EDXA spectra were collected. EDXA spectra collected from spot 1 (b) and spot 2 (c) and their elemental compositions are also reported.
(TIF)

S3 Fig The SEM-EDXA analysis of a cross sectioned MAP granule coated with bulk ZnO. The figure shows (a) scanning electron microscopy image of a cross sectioned MAP granule coated with bulk ZnO particles illustrating inner granule and coated surface of BulkMAP granule. The EDXA spectra collected from (b) spot 1 at the surface of coated granule and (c) spot 2 located in the core of granule as well as elemental composition at these points are shown.
(TIF)

S4 Fig The SEM-EDXA analysis of the surface of commercial urea granule. The figure illustrates (a) SEM image of the surface of commercial urea granule which was used in the experiment and (b) EDXA spectra collected from the point specified using a cross on the SEM image and elemental composition of the scanned point of interest.
(TIF)

S5 Fig The SEM-EDXA analysis of a cross-sectioned urea granule coated with bulk ZnO. The figure shows (a) the scanning electron microscopy image of a cross sectioned urea granule coated with bulk ZnO particles. The dark grey area at the right side of the image shows inner granule. Distribution of bulk ZnO particles at the surface of the granule can be observed at the left side of the image. The EDXA spectra collected from (b) points of interest at the surface of granule (spot1) and (c) inner granule (spot 2) to identify elemental compositions at these points.
(TIF)

S6 Fig The SEM-EDXA analysis of a urea granule coated with ZnO nanoparticles. The figure illustrates (a) SEM image of the surface of a urea granule coated with ZnO nanoparticles. The EDXA collected from (b) spot 1 and (c) spot 2 and the elemental compositions of aforementioned points of interest are reported in tables.
(TIF)
