# Supplementary figures and images for: Fate of Zinc Oxide Nanoparticles Coated onto Macronutrient Fertilizers in an Alkaline Calcareous Soil
Source: PLoS One. 2015 May 12;10(5):e0126275. doi: 10.1371/journal.pone.0126275 (PMC4428626; doi:10.1371/journal.pone.0126275)

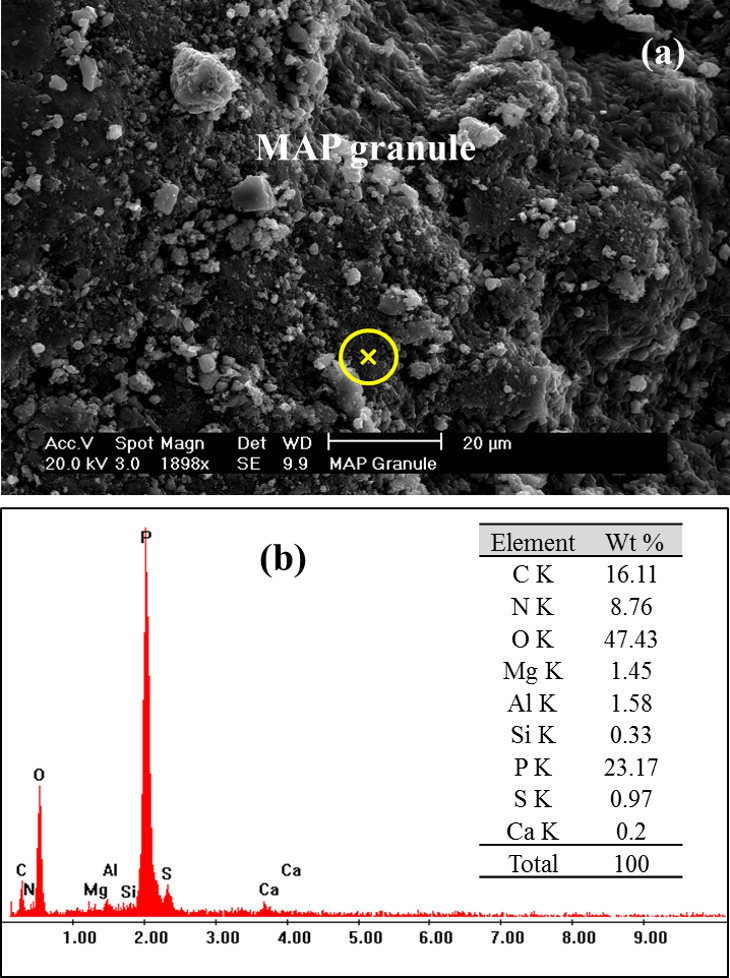

Supplement: S1 Fig — This figure shows (a) scanning electron microscopy (SEM) image of the surface of commercial mono ammonium phosphate (MAP) fertiliser granule used in the experiment and (b) EDXA spectrum that is collected from the point at the surface of the granule indicated by a cross on the SEM image. Elemental composition of the point of interest is reported in the table. (TIF) [file pone.0126275.s002.tif]

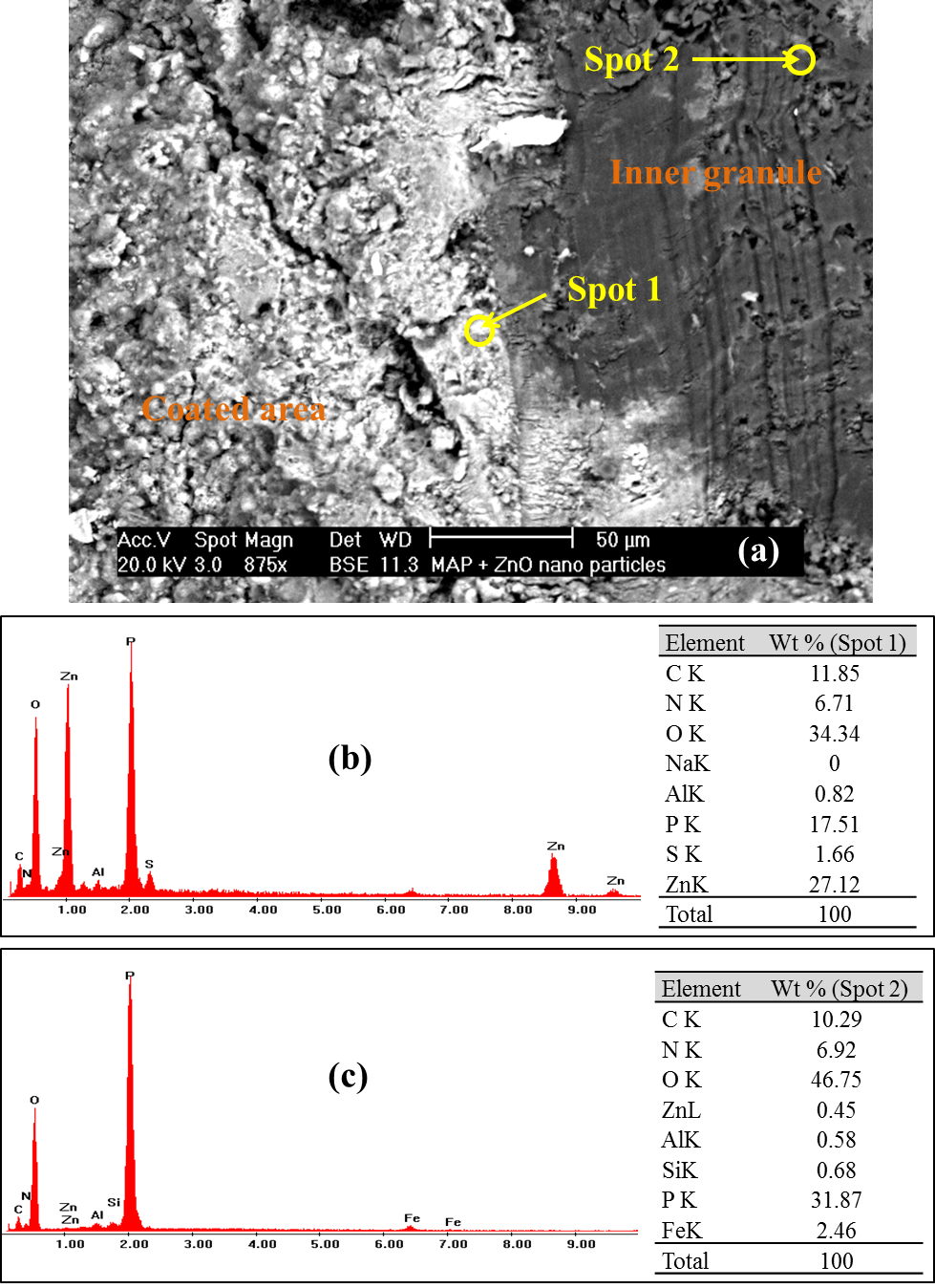

Supplement: S2 Fig — The figure illustrates (a) coated area (left) and inner granule (right) as well as the spots from which EDXA spectra were collected. EDXA spectra collected from spot 1 (b) and spot 2 (c) and their elemental compositions are also reported. (TIF) [file pone.0126275.s003.tif]

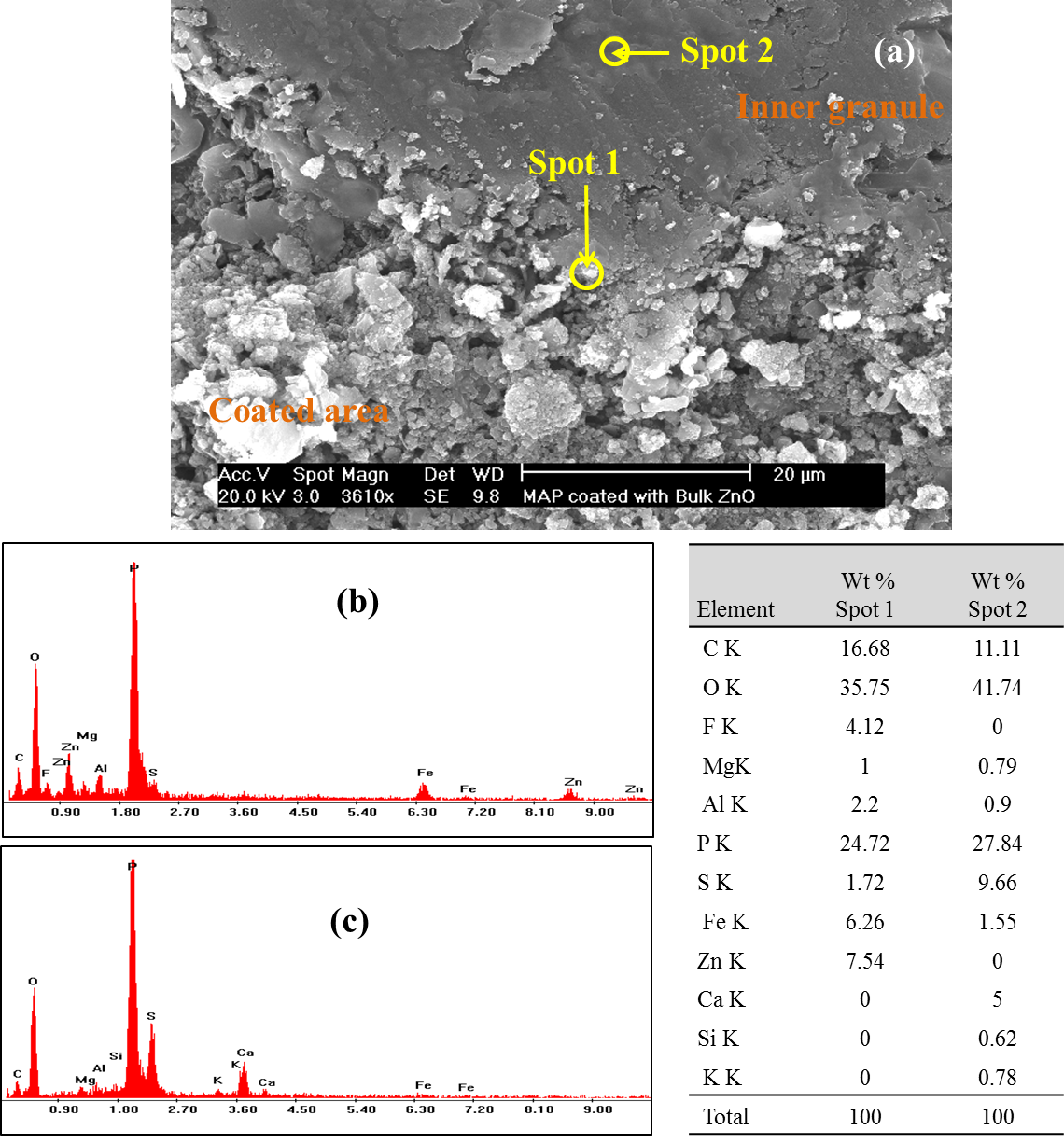

Supplement: S3 Fig — The figure shows (a) scanning electron microscopy image of a cross sectioned MAP granule coated with bulk ZnO particles illustrating inner granule and coated surface of BulkMAP granule. The EDXA spectra collected from (b) spot 1 at the surface of coated granule and (c) spot 2 located in the core of granule as well as elemental composition at these points are shown. (TIF) [file pone.0126275.s004.tif]

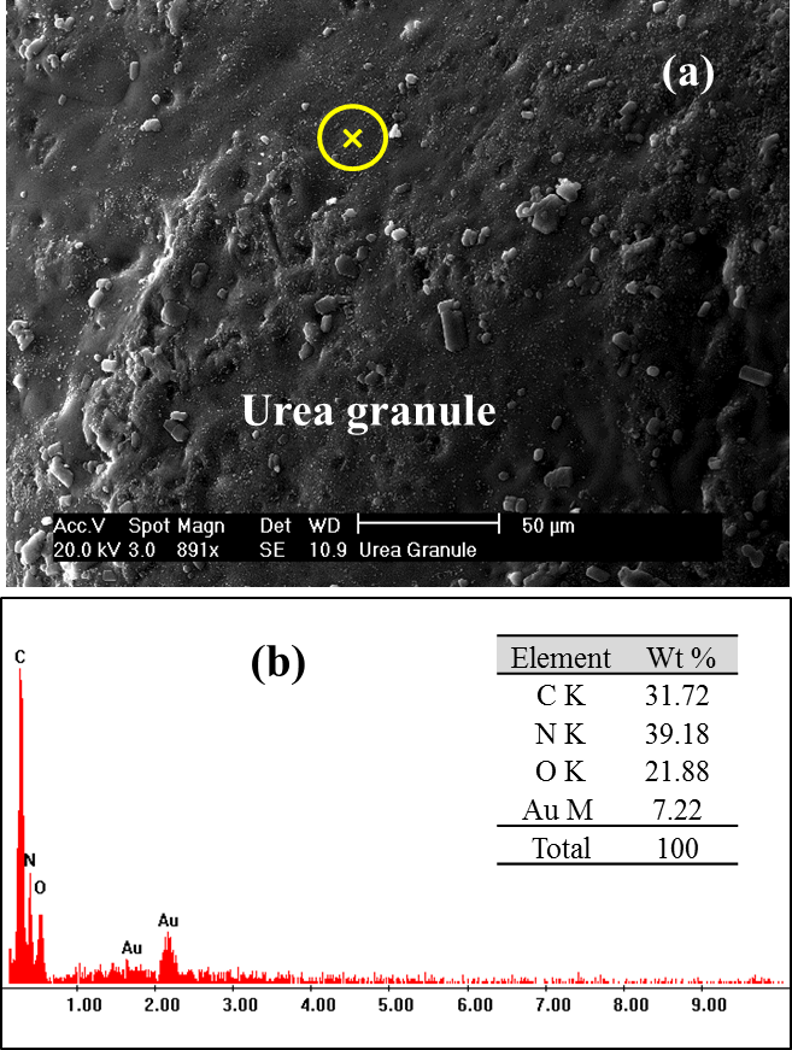

Supplement: S4 Fig — The figure illustrates (a) SEM image of the surface of commercial urea granule which was used in the experiment and (b) EDXA spectra collected from the point specified using a cross on the SEM image and elemental composition of the scanned point of interest. (TIF) [file pone.0126275.s005.tif]

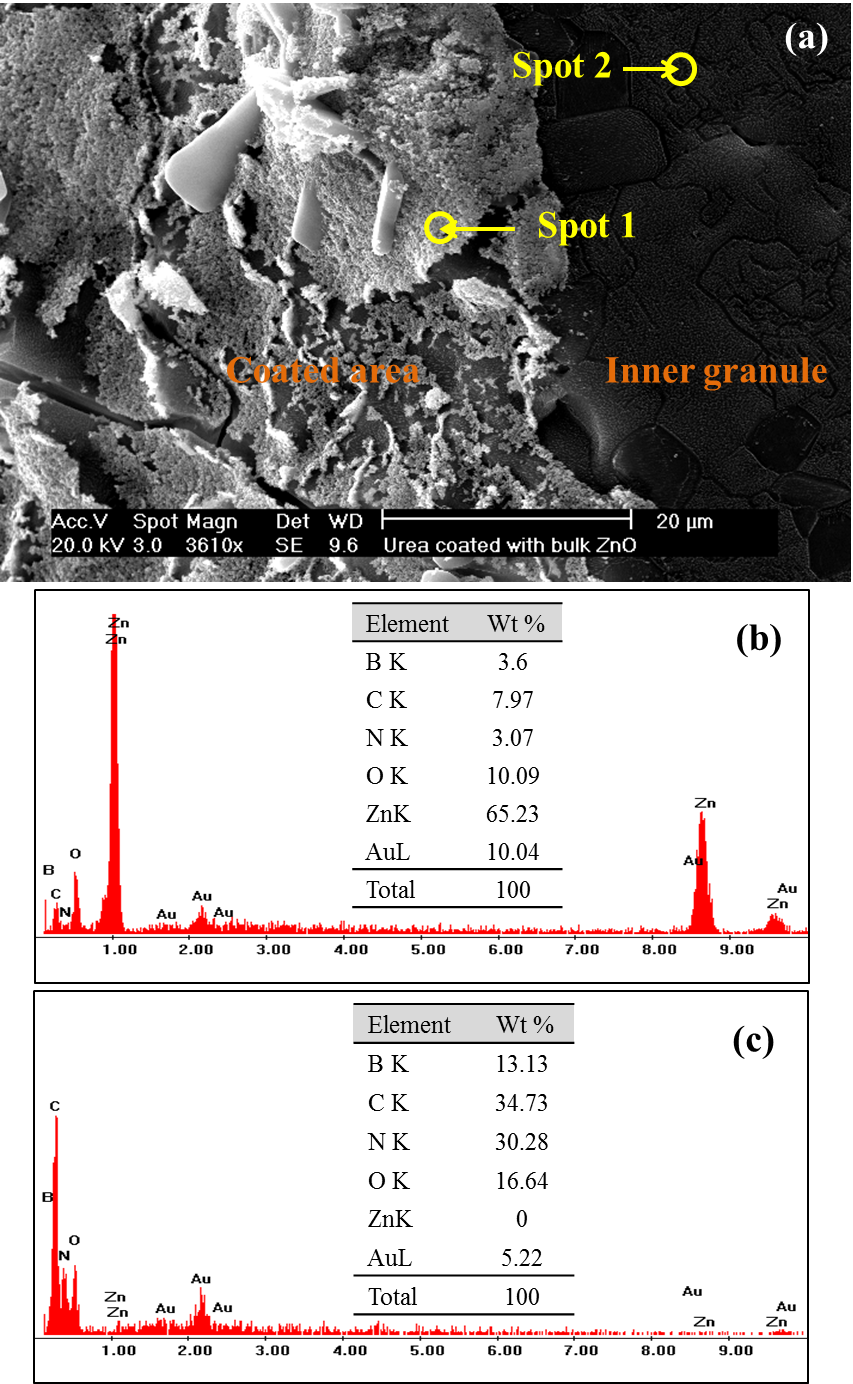

Supplement: S5 Fig — The figure shows (a) the scanning electron microscopy image of a cross sectioned urea granule coated with bulk ZnO particles. The dark grey area at the right side of the image shows inner granule. Distribution of bulk ZnO particles at the surface of the granule can be observed at the left side of the image. The EDXA spectra collected from (b) points of interest at the surface of granule (spot1) and (c) inner granule (spot 2) to identify elemental compositions at these points. (TIF) [file pone.0126275.s006.tif]

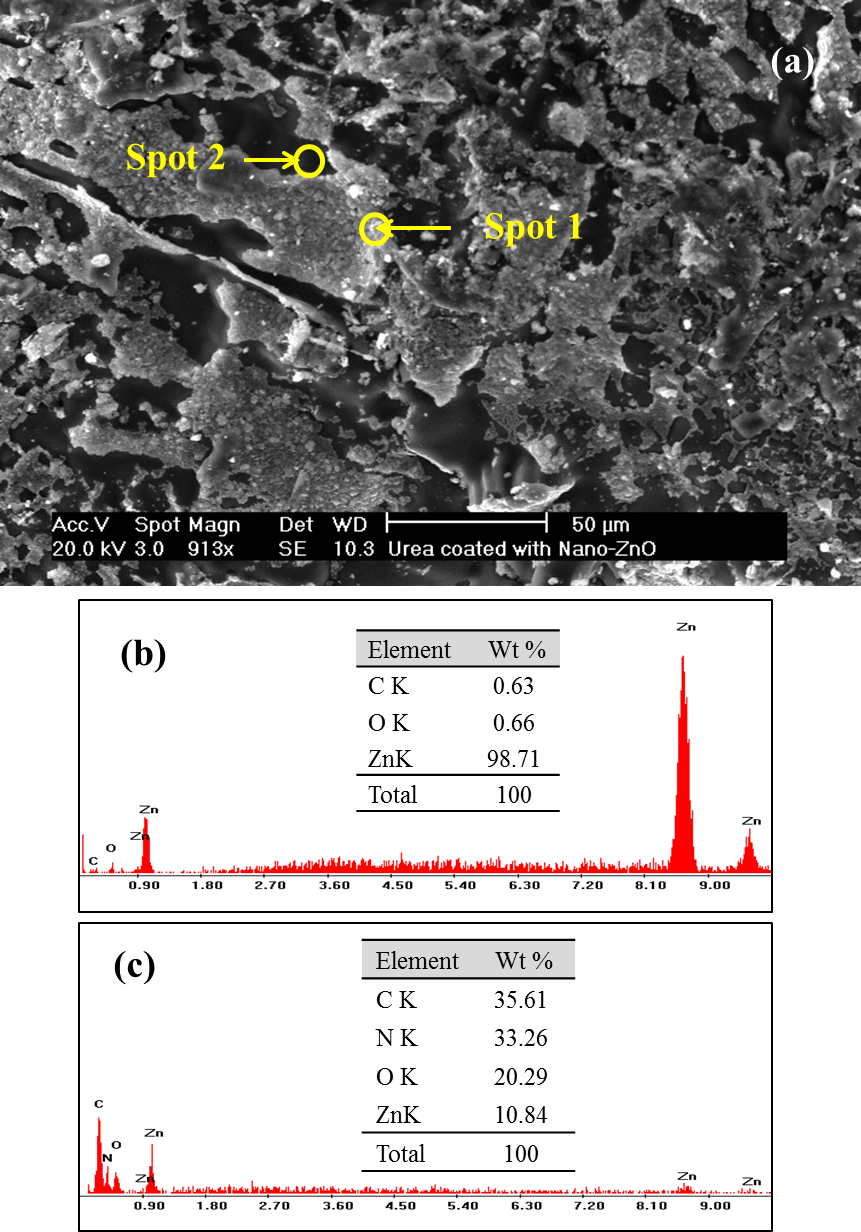

Supplement: S6 Fig — The figure illustrates (a) SEM image of the surface of a urea granule coated with ZnO nanoparticles. The EDXA collected from (b) spot 1 and (c) spot 2 and the elemental compositions of aforementioned points of interest are reported in tables. (TIF) [file pone.0126275.s007.tif]
